# A case report of tuberculous meningitis resulting in irreversible visual impairment due to delayed diagnosis

**DOI:** 10.1002/ccr3.9334

**Published:** 2024-08-19

**Authors:** Erika Lee‐Yu Lin, Sai Anil Gulhane, Majety Sameer Kumar, Sai Teja Lakkamaneni, Prudhvi Lekkala

**Affiliations:** ^1^ School of Medicine Xiamen University Xiamen P.R. China; ^2^ Andhra Medical College Andhra University Visakhapatnam Andhra Pradesh India; ^3^ Medical College Kyrgyz Russian Slavic University Bishkek Kyrgyzstan

**Keywords:** irreversible vision loss, tuberculosis, tuberculosis and meningitis, Ventriculo‐Peritoneal shunt

## Abstract

**Key Clinical Message:**

Prompt diagnosis of tuberculous meningitis (TBM) is crucial to prevent severe complications like cranial nerve involvement and irreversible visual impairment. Early suspicion and intervention are essential, especially in tuberculosis‐endemic regions. Rapid initiation of anti‐tuberculosis therapy and vigilant monitoring for complications, such as hydrocephalus, improve patient outcomes and prevent long‐term disabilities.

**Abstract:**

This case study provides a comprehensive overview of the difficulties associated with predicting and managing tuberculous meningitis (TBM). The predictive aspect is hindered by the subacute nature of TBM, featuring a prodromal phase lasting 7–10 days, followed by manifestations like severe headaches, altered mental status, stroke, hydrocephalus, and cranial neuropathies. Additionally, vision loss is a disabling complication. All components of the visual pathway, especially the optic nerve and optic chiasma, are frequently and dominantly affected. While antibiotics can promptly resolve meningitis in most cases, approximately 10% of infections progress to chronic meningitis, with tuberculosis meningitis being the most common form. Our patient initially presented with nonspecific symptoms, which later evolved into symptoms that indicate viral meningitis and was started on empirical therapy. Subsequently, due to clinical suspicion of tuberculosis meningitis and persistent symptoms despite treatment, she was placed in anti‐tuberculosis therapy (ATT) but unfortunately developed complications such as hydrocephalus and blindness. To address the hydrocephalus, a Ventriculo‐Peritoneal shunt was implanted. Despite delayed treatment and diagnosis, most of her symptoms resolved except for blindness, for which there was only partial recovery.

## 1 INTRODUCTION

Tuberculous meningitis (TBM) is a common manifestation of central nervous system tuberculosis. It is associated with primary infection, usually localized in the lungs with dissemination to the lymph nodes. The bacteria then travel through the bloodstream to the meninges, resulting in a high bacterial count and the formation of microtubercles. TBM develops when these microtubercles rupture. Vision loss is a disabling complication of tuberculous meningitis.^1^

In developing countries, this infection is more prevalent among infants and toddlers, while in developed countries, adults are more commonly affected due to reactivation of dormant TB infections.

Ophthalmic complications are common in TBM patients, including optic neuritis, optic atrophy, and papilloedema due to increased intracranial pressure. Vision impairment could result from the disease process or anti‐tuberculosis treatment (ATT).^2^

## 2 CASE PRESENTATION

Here, we present a clinical case report of a 35‐year‐old female presenting to the ER with a 10‐day history of fever, chills, rigor, transient neck stiffness, severe headache, myalgia, arthralgia, and non‐blood‐stained vomiting episodes. She was also noted to be 7 month postnatal. Physical examination revealed bilateral cervical lymphadenopathy and increased sensitivity to light.

**FIGURE 1:**
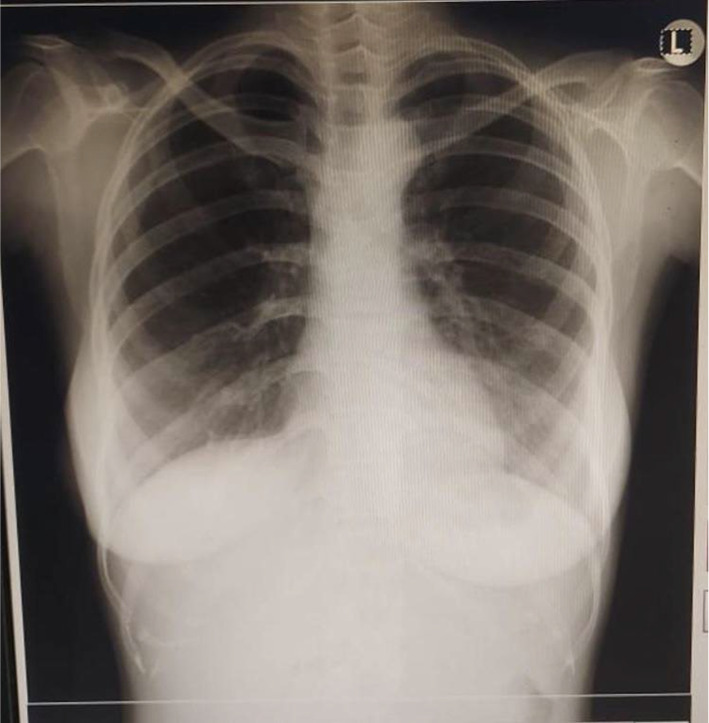
Chest x‐ray of patient.

**FIGURE 2:**
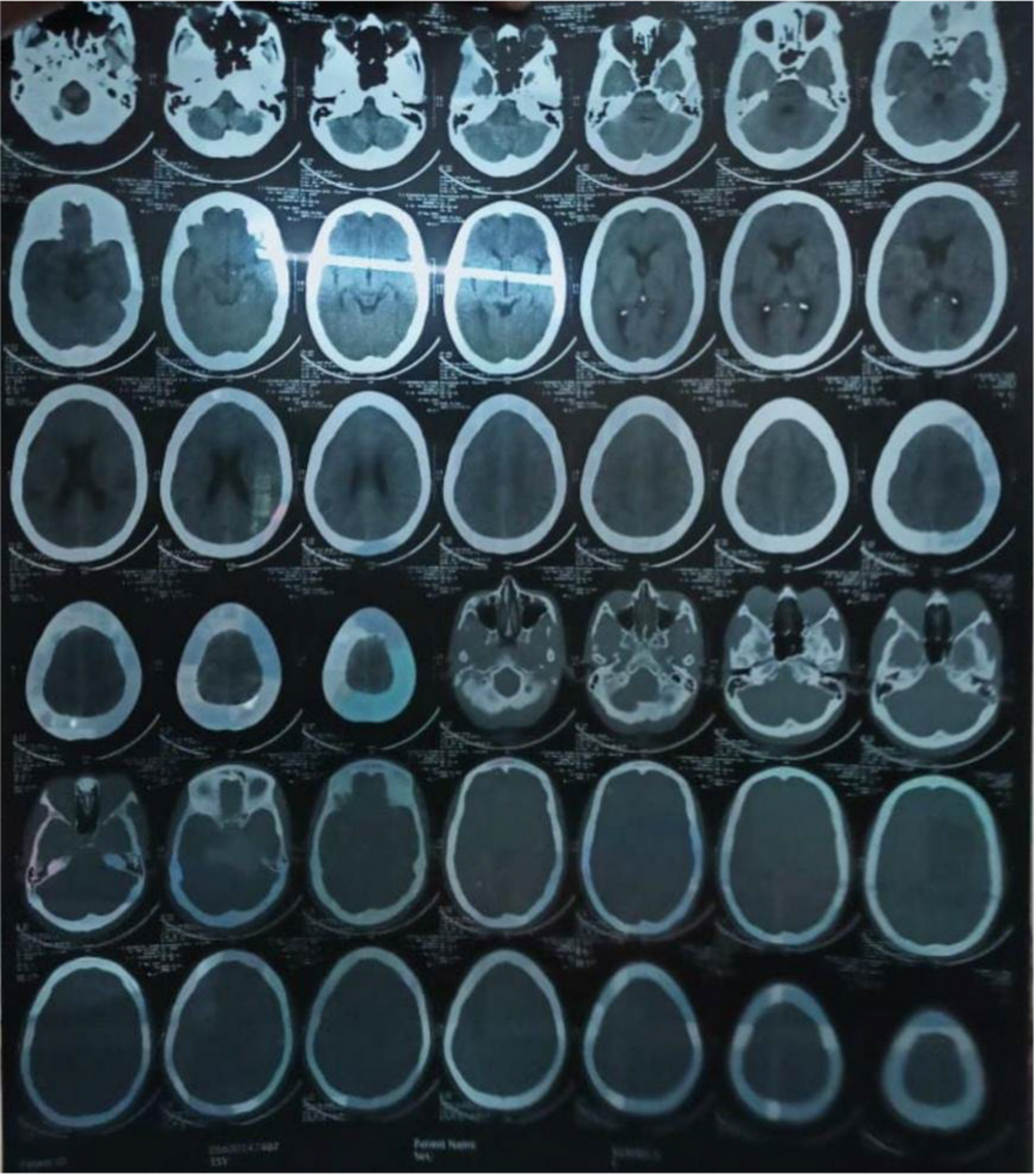
Axial sections of CT brain.

**FIGURE 3:**
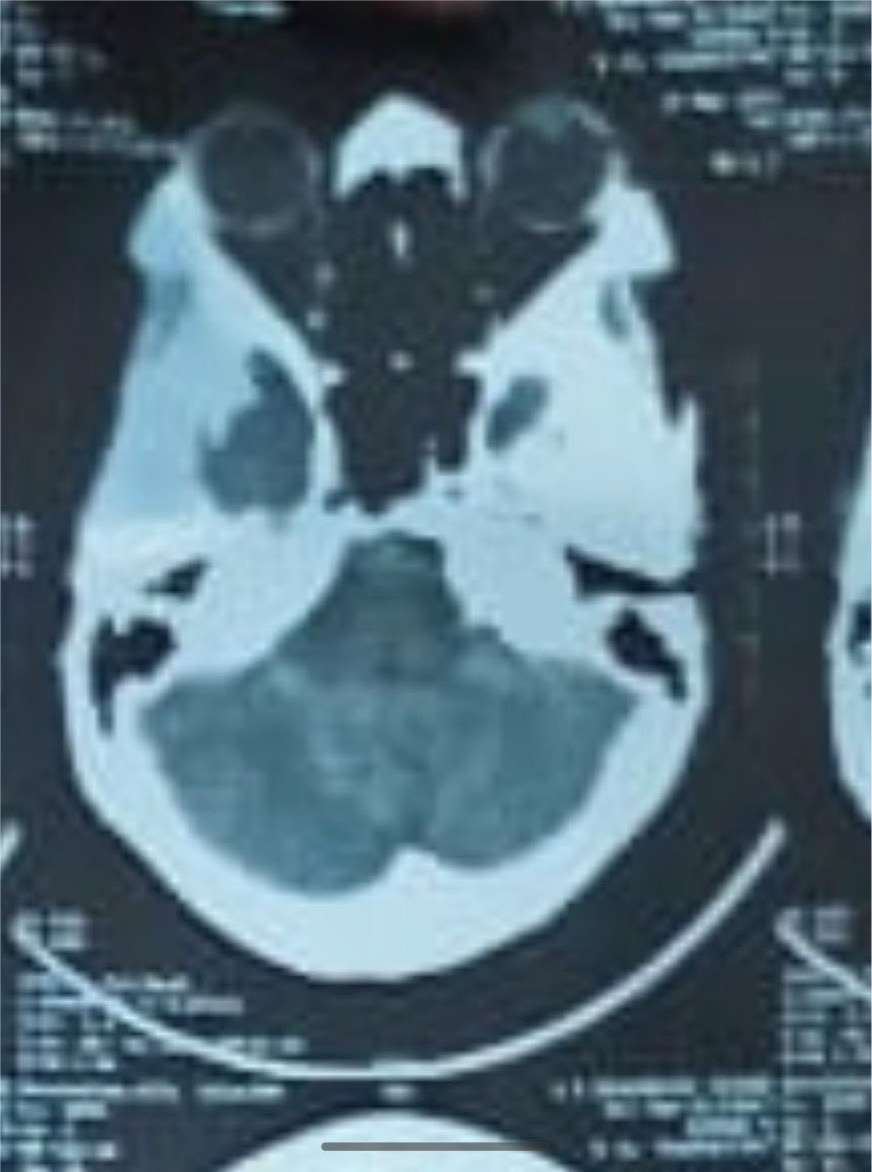
Axial section of CT Brain showing base of skull.

Initial management included empirical antibiotics and symptomatic treatment. In the suspicion of infection, various diagnostic tests were conducted, including blood and urine cultures, malaria antigen test, viral profiles (HBV, HCV, HIV, and dengue), sputum CBNAAT for Mycobacterium, urinalysis (Table 1), and a chest x‐ray (Figure 1). Additional investigations comprised a CT Brain (Figures 2 and 3) and fine needle aspirate cytology (FNAC) of cervical lymph nodes.

**TABLE 1:** Table containing all the diagnostic information of the patient.

Microbiology Report		
Specimen	Results	Notes
Blood culture	Negative for bacterial growth	
Urine culture	Negative for bacterial growth	No parasites, ova
Malaria antigen test (blood specimen)	Negative	
Viral profiles (HBV, HCV, HIV, dengue, etc.)	Negative	
Sputum CBNAAT (mycobacterium)	Negative	
CUE (complete urinalysis examination)	Normal CUE: yellowish, clear, no protein, 0–1 cells	
Chest x‐ray	No abnormalities	
CT brain	All structures, spaces, regions normal and no evidence of hemorrhage/infarct No extra axial collections Ventricular system is prominent	Technique: Serial axial sections 5 mm thickness of posterior fossa
MRI	Enhanced basal exudates with hydrocephalus Ischemic changes in vessels Thickening of oculomotor nerve Left lentiform nucleus restricted diffusion	

Following inconclusive test results and persistent clinical deterioration, a provisional diagnosis of viral fever was made, and the patient was placed on appropriate therapy. Unfortunately, there was no improvement in her condition. On the 10th day of admission, she developed altered sensorium and generalized weakness, prompting an immediate transfer to the intensive care unit.

In the ICU, a lumbar puncture was performed, and cerebrospinal fluid (CSF) was obtained for further studies. However, CSF cultures for bacteria and smear for acid‐fast bacilli (AFB), CBNAAT/Gene Xpert yielded negative results. The biochemical profile of CSF, including glucose, protein, albumin, and chloride, did not indicate any specific etiology. The patient reported blurring of vision and exhibited signs of Lateral rectus palsy during examination, highlighting the complexity of the case.

Following a comprehensive reevaluation, a diagnosis of tuberculous meningitis was established based on factors such as the endemic nature of the disease in the population, and the exclusion of other potential causes. The patient was promptly initiated on anti‐tuberculosis treatment (ATT). MRI of the brain was conducted, revealing enhancing basal exudates with hydrocephalus, further confirming the diagnosis of TB.^3^ The MRI also displayed small vessel ischemic changes, thickening, and enhancement of the occulomotor nerve, along with small areas of restricted diffusion in the left lentiform nucleus and thalamus, suggestive of subacute infarcts.

Additionally, a HRCT chest (high‐resolution CT) uncovered multiple nodular infiltrates with septal thickening in bilateral lung parenchyma. Patchy atelectasis was observed in the bilateral lower lobes, accompanied by a few enlarged sub‐carinal lymph nodes. These findings on imaging collectively supported the diagnosis of Tuberculous Meningitis, indicating the systemic impact of the infection.

Upon re‐examination, it was discovered that the patient had experienced a complete loss of vision, later confirmed by an ophthalmology consultation as blindness secondary to tuberculous meningitis. This visual impairment was attributed to hydrocephalus resulting in optic atrophy and optic chiasmatic arachnoiditis.^2^

**FIGURE 4:**
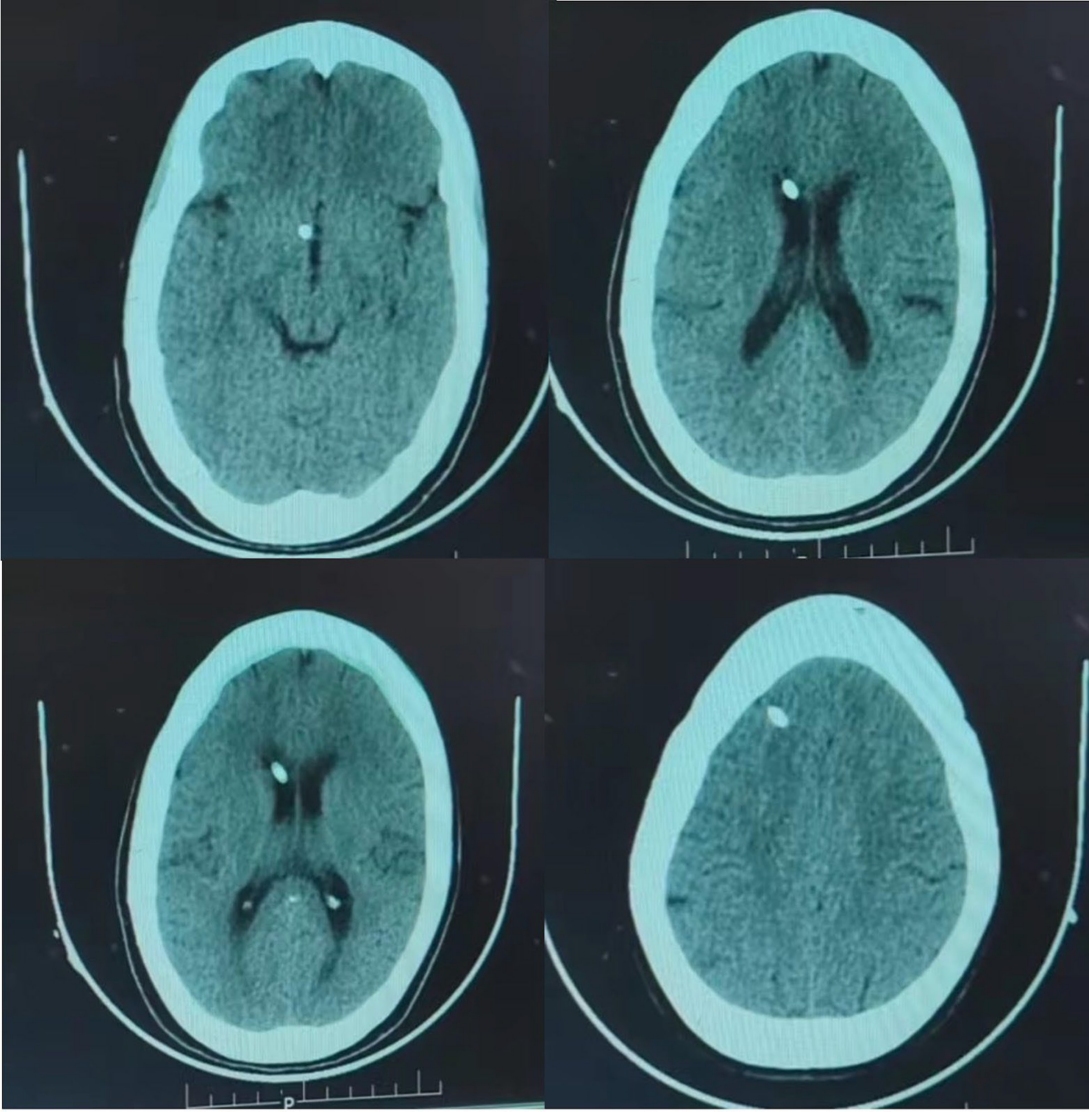
Axial sections of CT brain of our patient showing potency of VP shunt 9 months post surgery.

Subsequently, a referral to neuro‐surgery was made, and a plan was devised to address the hydrocephalus by placing a Ventriculo‐Peritoneal Shunt (VP shunt). Following a thorough pre‐operative evaluation, the VP shunt was successfully placed to alleviate hydrocephalus (Figure 4). Additionally, an Ommaya Reservoir was implanted in the patient's right fronto‐temporal region without encountering any post or intraoperative complications. These interventions aimed to manage the underlying causes contributing to the patient's visual impairment.

After initiating ATT and placing the VP shunt, the patient experienced positive changes in her clinical course, with a reduction in fever, generalized weakness, and altered sensorium. However, substantial improvement in vision was not evident until the 10th post‐operative day when the patient began to notice changes in light and observe object movements.

In the subsequent weeks, the 35‐year‐old patient stabilized, leading to her discharge from the hospital. The continuation of ATT and regular outpatient follow‐ups were recommended. While the patient's general health fully recovered, her vision did not reach a satisfactory level during the follow‐up visits in the ensuing months. Ophthalmology consultations revealed an increasing prominence of Optic Atrophy in the examinations, underscoring the persistent challenges associated with visual impairment despite overall health improvement.

## 3 DIAGNOSIS

The diagnosis of this disease is challenging due to its insidious onset, diverse clinical presentation, and difficulty in detecting Mycobacterium tuberculosis in cerebrospinal fluid (CSF).^14^ The CSF acid fast smear is known to have a low sensitivity of 20%–40%.

Although visualizing the mycobacteria in CSF, via a smear or culture, remains the gold standard for definitive diagnosis,^8^ these characteristic CSF findings also play a critical role in the diagnosis:

1. Lymphocytic‐predominant pleiocytosis. Total white cell counts are usually between 100 and 500cells/μL
2. Elevated protein levels, typically between 100 and 500 mg/dL.
3. Low glucose, usually less than 45 mg/dL or CSF:Plasma ratio <0.5.
4. CSF opening pressure‐increases (350–500 mm H_2_O).^9^

In our patient, we have faced difficulty in diagnosing the disease because of its insidious progression. In addition, the CSF analysis and the CSF smear both were inconclusive. Therefore, the diagnosis was made based on the chronic nature of the signs and symptoms, clinical features and radio imaging.

The neuroimaging in tuberculous meningitis shows hydrocephalus due to blockage of CSF by basal enhancing exudates (non‐obstructive /communicating), ring or disc shaped enhancing lesions of tuberculomas, vasculitic basal ganglia infarcts due to the blood vessels that supply the brain lie along basal cisterns.^13^ In presence of infective exudates, vasculitis may also occurs; leptomeningeal enhancement along sylvian fissures and ependymitis may be present. The MRI brain scan of this 35‐year old patient revealed an enlarged ventricular system indicating hydrocephalus and enhancing exudates along the basal cisterns, suggestive of tubercular etiologies: small vessel ischemic changes, thickening and enhancement of oculomotor nerve(CN 3), small areas of restricted diffusion in left lentiform nucleus (subacute infarcts) and left thalamus.

## 4 COMPLICATIONS

The most prevalent complication of tuberculous meningitis is hydrocephalus, which is abnormal retention of CSF in the ventricles that can lead to increased intracranial pressure. It is usually a late complication and is characterized by granulomatous inflammatory exudate obstructing CSF flow. In this report, the patient's condition worsened due to hydrocephalus which ultimately led to the loss of her visual acuity.

Hydrocephalus can impact vision through two mechanisms: causing damage to the optic nerve, and affecting the nerves that control eye muscles. Elevated ICP exerts pressure on the brain and meninges, compressing the optic nerve directly and reducing blood flow to it. The diminished nutrient and oxygen supply to the optic nerve leads to impaired function and swelling, known as “papilledema.” Papilledema‐induced damage can result in diminished vision, reduced color perception, and visual field loss.^10^

The impairment of cranial nerves can also occur, specifically the third, fourth, and sixth cranial nerves which are responsible for eye position and movement, due to hydrocephalus. This impairment can lead to issues like eye misalignment (strabismus) or double vision (diplopia). In infants, a phenomenon known as the “sunset eye sign” can also manifest, where the eyes are fixed in a downward and outward position. There are also few other complications which are infrequent but can occur, they are stroke, seizures, spread to spinal cord, radiculomyelitis, SIADH, and optochiasmatic arachnoiditis.^11^

Blindness in TB can also be caused by optochiasmatic arachnoiditis (OCA) resulting in ischemia of the optic chiasm and optic nerve and optochiasmal tuberculoma.^2^

## 5 TREATMENT

The current ATT involves a combination therapy comprising an intensive 2‐month period of isoniazid, rifampin, pyrazinamide, and ethambutol. This is followed by a continuation period of 4 months with isoniazid and rifampin. Directly observed therapy (DOT) is strongly recommended to ensure adherence to this complex regimen and prevent the spread or metastasis of TB to other organs. Statistically, treatment is successful in approximately 85% of patients following the 6‐month therapy.^7^, ^8^, ^12^

However, even after treatment, patients may have an existing lung disease and a decreased life expectancy. In addition, multidrug‐resistant TB treatment has been known to decline by 15%, meaning only a third of patients who are known to be needing this treatment are gaining access.^8^

**FIGURE 5:**
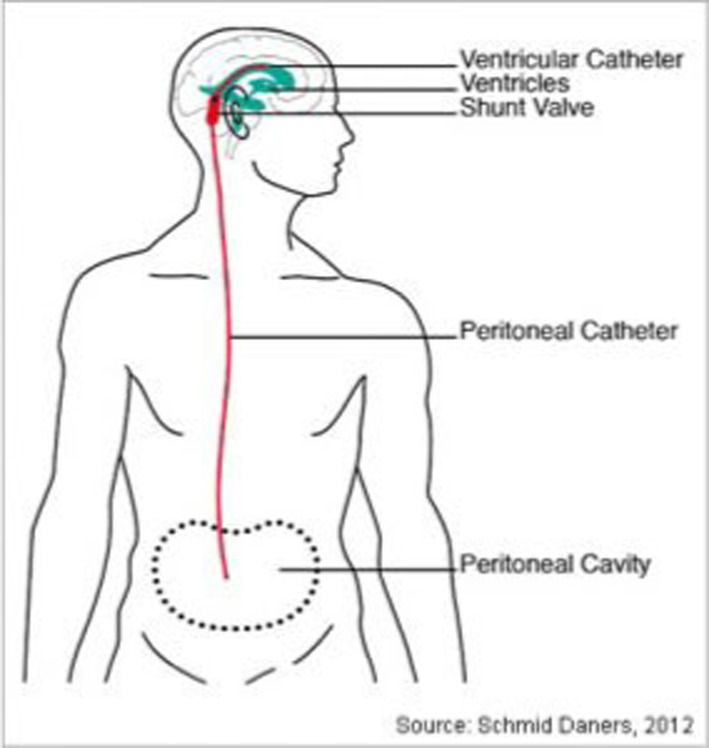
Sketch of a ventriculoperitoneal shunt involves draining cerebrospinal fluid from a lateral ventricle to the peritoneum in a patient.^15^

**FIGURE 6:**
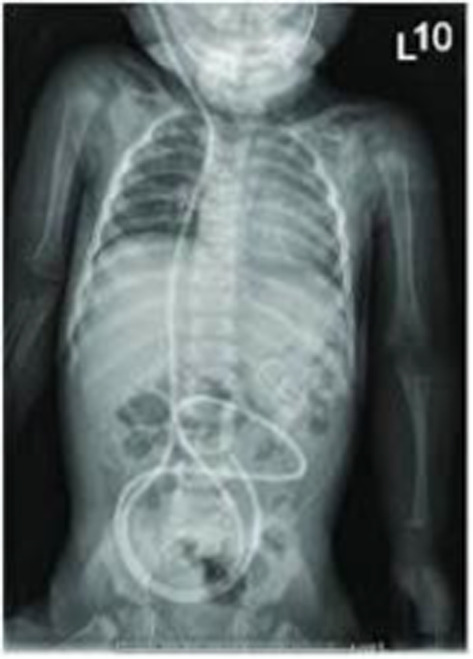
Peritoneal portion of the VP shunt.^16^

After a thorough preoperative examination the patient in case was taken up for ventriculoperitoneal shunt placement (VP shunt). A VP shunt is comprised of two catheters which diverts the extra CSF from the ventricles into the peritoneal space of the abdomen. The CSF diverted to the peritoneal space is reabsorbed into the bloodstream and eventually discharged through normal urination (Figures 5 and 6).

## 6 CONCLUSION

Tuberculous meningitis is considered rare due to its atypical early symptoms and delayed laboratory examinations. Early identification, diagnosis, and initiation of treatment is proven challenging, but given the seriousness of TBM, its neurological deficits, morbidity and mortality rates, rapid intervention is crucial. Despite these challenges, TBM remains the most common form of chronic central nervous system infection, underscoring the importance of advancing diagnostic and therapeutic approaches.

It is estimated that there are more than 2 billion people in the world who are in contact with tuberculosis, but only 10% will manifest into clinical diseases. The challenging diagnosis of tuberculosis meningitis is evident in our case study, given its atypical presentation and insidious progression. Reliable tests are lacking, emphasizing the crucial role of promptly diagnosing and initiating anti‐tuberculosis therapy to save the patient's vision and disease. While the current prescription involves a VP shunt considering the circumstances, it alone falls short in addressing the patient's condition, particularly regarding blindness.

## 7 DISCUSSION

Tuberculosis (TB), caused by Mycobacterium Tuberculosis, is an infectious disease widespread in the developing world. While it primarily affects the lungs through the inhalation of airborne droplets, it can manifest in various parts of the body. Common targets include the spine and brain, with the bacilli spreading from a primary lung infection to cause extrapulmonary manifestations such as tuberculous meningitis (TBM).

Meningitis, an inflammation of the meninges that encircles the spinal cord and brain, is generally caused by viral or bacterial infection. Tuberculous meningitis typically presents with a history of anorexia, fatigue, fever, malaise, and headache lasting 2 to 8 weeks. Additionally, approximately 40%–80% of patients may experience neck stiffness.^4^ The duration of the symptoms before the presentation may range from days to months. There are generally three stages to tuberculous meningitis.

- Stage I: alert without focal neurological deficits.
- Stage II: Glasgow coma score of 11–14 with focal neurological default.
- Stage III: Glasgow coma score of 10 or less, with/without focal neurological default.^5^

Although most cases of meningitis can resolve promptly with a course of antibiotics, few such as 10%, may progress to chronic meningitis.^6^ One of the most common causes of chronic meningitis is TBM.

Mycobacteria can cause disease in three forms as it migrates to the central nervous system:

1. Tuberculoma (space occupying lesion).
2. Basilar arachnoiditis.
3. Chronic meningitis which is usually due to rupture of subependymal focus of metastatic caseous lesion known as RICH'S focus. It can also occur because of disseminated hematogenous spread in which miliary shadows can be seen on chest x‐ray.

This infection is more prevalent in the developing world, with a higher incidence in children of age 0–4 years.MTB in children. By contrast, in the developed world, TBM is more often seen in adults who experience the reactivation of TB. Other immunocompromised individuals such as chronic steroid use, diabetes mellitus, and chronic alcoholism carry the same risk of developing TBM. The highest correlation remains with HIV co‐infection, with reports that these patients are 5 to 10 times more likely to develop CNS disease.^1^ Post‐ partum TB meningitis is not very clear but can be associated with rapid postpartum immune reconstitution causing flare up of underlying disease. This is again more common in HIV positive patients. Pregnant and postpartum women could also be at a higher risk of developing TB meningitis associated neurological deficits attributing to their hypercoagulable state. However, studies show postpartum TB meningitis manifests in earlier weeks; hence, is ruled out in our patient who is 7 months post natal.^7^

## AUTHOR CONTRIBUTIONS

**Erika Lee‐Yu Lin:** Data curation; formal analysis; investigation; writing – original draft; writing – review and editing. **Sai Anil Gulhane:** Conceptualization; project administration; writing – original draft; writing – review and editing. **Majety Sameer Kumar:** Project administration; supervision; validation; visualization; writing – original draft; writing – review and editing. **Sai Teja Lakkamaneni:** Conceptualization; methodology; project administration; supervision. **Prudhvi Lekkala:** Conceptualization; data curation; formal analysis; supervision.

## FUNDING INFORMATION

No funding received.

## CONFLICT OF INTEREST STATEMENT

The authors declare no conflict of interest.

## ETHICS STATEMENT

In our university, ethics approval was not required for case reports and case series.

## CONSENT

Written informed consent was obtained from the patient to publish this report in accordance with the journal's patient consent policy.

## Data Availability

The data supporting this case report is available from the corresponding author upon reasonable request.
